# Sub-single exciton optical gain threshold in colloidal semiconductor quantum wells with gradient alloy shelling

**DOI:** 10.1038/s41467-020-17032-8

**Published:** 2020-07-03

**Authors:** Nima Taghipour, Savas Delikanli, Sushant Shendre, Mustafa Sak, Mingjie Li, Furkan Isik, Ibrahim Tanriover, Burak Guzelturk, Tze Chien Sum, Hilmi Volkan Demir

**Affiliations:** 1 Department of Electrical and Electronics Engineering, Department of Physics, UNAM-Institute of Materials Science and Nanotechnology, Bilkent University, Ankara, 06800 Turkey; 2 Luminous! Centre of Excellence for Semiconductor Lighting and Displays, School of Electrical and Electronic Engineering, School of Physical and Mathematical Sciences, School of Materials Science and Engineering, Nanyang Technological University, Singapore, 639798 Singapore; 3 School of Physical and Mathematical Sciences, Nanyang Technological University, Singapore, 639798 Singapore; 4 Advanced Photon Source, Argonne National Laboratory, Lemont, IL 60439 USA

**Keywords:** Semiconductor lasers, Semiconductor lasers

## Abstract

Colloidal semiconductor quantum wells have emerged as a promising material platform for use in solution-processable lasers. However, applications relying on their optical gain suffer from nonradiative Auger decay due to multi-excitonic nature of light amplification in II-VI semiconductor nanocrystals. Here, we show sub-single exciton level of optical gain threshold in specially engineered CdSe/CdS@CdZnS core/crown@gradient-alloyed shell quantum wells. This sub-single exciton ensemble-averaged gain threshold of (*N*_g_)≈ 0.84 (per particle) resulting from impeded Auger recombination, along with a large absorption cross-section of quantum wells, enables us to observe the amplified spontaneous emission starting at an ultralow pump fluence of ~ 800 nJ cm^−2^, at least three-folds better than previously reported values among all colloidal nanocrystals. Finally, using these gradient shelled quantum wells, we demonstrate a vertical cavity surface-emitting laser operating at a low lasing threshold of 7.5 μJ cm^−2^. These results represent a significant step towards the realization of solution-processable electrically-driven colloidal lasers.

## Abstract (web summary)

Colloidal quantum wells are highly promising for applications of solution-processed lasers, but their performance is limited by multi-excitonic nature of the materials. Here, the authors demonstrate optical gain in graded alloy core/shell CdSe/CdS@CdZnS quantum wells at less than one exciton per particle resulting in ultralow thresholds.

## Introduction

Solution-processed semiconductor nanocrystals offer a versatile and promising gain medium for light-amplification applications^1–4^ owing to their broad spectral tunability^1,5,6^, low-cost production and flexibility of using them in a broad range of matrices^1,4^. As a consequence, the utilization of colloidal semiconductor quantum dots (CQDs) as a gain medium has been experiencing a tremendous growth in the last few decades. In addition, alternative nanoemitters, e.g., semiconductor nanorods^7^ and perovskite nanocrystals^8,9^, have recently emerged as an auspicious gain material for stimulated emission and lasing. Yet another promising candidate, colloidal semiconductor quantum wells (CQWs), the so-called nanoplatelets (NPLs)^10–12^, have been shown to be excellent in optical gain applications thanks to their giant oscillator strength^13^ and ultralarge modal gain coefficient^14^. Using different heterostructures of NPLs allows for ultralow-amplified spontaneous emission (ASE) thresholds^10,12^. However, their gain performance still suffers from nonradiative Auger recombination, wherein the released energy from recombination of the electron-hole is transferred to a third carrier. In common II–VI semiconductor nanocrystals, because of the non-unity degeneracy of the electron and hole states involved in emission, light amplification requires an ensemble-averaged number of excitons per nanocrystal greater than one ((*N*) > 1), thereupon multi-excitonic Auger recombination strongly affects the dynamics of the carriers.

Previously, various efforts have been reported to tackle the issue of the fast Auger decay in nanocrystals including the smoothing of their confinement potential^15^ and introducing the optical gain in single-exciton regime^2,7,16^. These methods have been shown to effectively enhance the optical gain performance of CQDs either by reducing influence or inactivating of the Auger process. On the other hand, in atomically flat CQWs, significant reduction in ASE and lasing threshold have previously been obtained in core/shell^12^ and core/crown^10,11^ heterostructures. Nonetheless, the demonstrated approaches have not addressed the fundamental issue of the optical gain in CQWs where the multi-exciton ((*N*) > 1) nature of the light amplification results in fast decay of the carriers by a few hundred of picoseconds timescale^17,18^ as a result of the Auger process. One way of inactivating of Auger recombination in CQWs is to employ the concept of single-exciton gain mechanism for optical amplification as formerly demonstrated in various heterostructures of the CQDs^2,7,16^. Particularly, a finite Stokes shift of the stimulated emission with respect to absorption peak can be employed to achieve optical gain in sub-single exciton regime where the Auger process is mostly inactivated^2,19^. As presented in our previous work^20^, the Stokes shift is increased with the increasing Zn content in the shell. This shift is finally maximized in CdSe/CdS@ZnS core/crown@shell heterostructure. As a result, the Zn concentration in our CdSe/CdS/Cd_1-x_Zn_x_S core/crown@alloyed-shell CQWs can be modified to tune the Stokes shift to engineer the optical gain in these CQW heterostructures.

In the current study, we have exploited the finite Stokes shift as a tool for achieving optical gain threshold in sub-single exciton regime ((*N*) < 1) in quasi-type-II CdSe/CdS@Cd_1-x_Zn_x_S core/crown@gradient-alloyed shell (C/C@GS) CQWs, which presents an important step towards the evolution of semiconductor CQW lasers. In addition, these core/crown@gradient-alloyed shell CQWs offer a promising solution for suppression of the Auger process with their smooth confinement potential. We demonstrated an exceptionally low stimulated emission threshold of ~820 nJ cm^−2^, corresponding to an average number of e–h pairs of 0.84 per NPL, which is also fully supported by nonlinear absorption measurements through ultrafast transient absorption spectroscopy. Sub-single exciton optical gain regime is also confirmed by linear dependence of the normalized absorption changes. The extremely large absorption cross-section (5.06 × 10^−13^ cm^2^) of our engineered NPLs, accompanied by an extremely large net modal gain coefficient of ~1960 cm^−1^, and a long net optical gain lifetime of ~830 ps result in such ultralow optical gain thresholds and lead to record-long stable ASE. Employing specifically engineered core/crown@gradient-alloyed shell heterostructures, we present a linearly polarized single-mode lasing from a vertical-cavity surface-emitting laser enabling a record low lasing threshold of ~7.46 μJ cm^−2^.

## Results

**Fig. 1:**
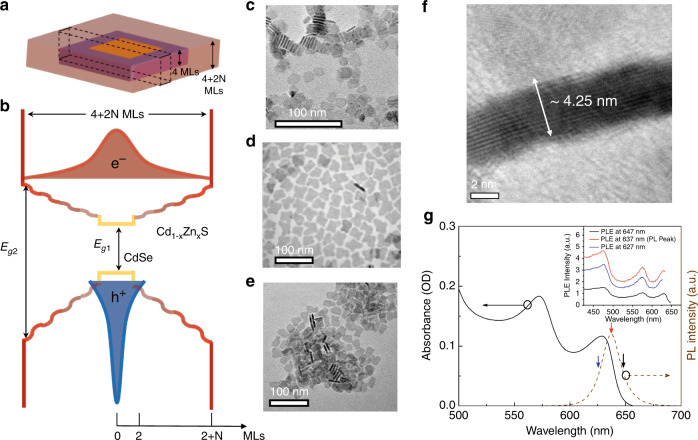
Structure and characterization of the synthesized CQWs. **a** Schematic illustration of C/C@GS of CdSe/CdS@Cd_1-x_Zn_x_S NPLs synthesized by using interfacial grading of shell in the vertical dimension as detailed in the text. **b** An approximate energy band diagram of the graded confinement potential in C/C@GS CQWs (based on band offset of bulk semiconductor^1^) along with a schematic representation of the spatial distribution of electron (e^−^) and hole (h^+^) wavefunctions. Here, E_g1_ ≈ 1.75 eV and E_g2_ ≈ 2.7 eV for 4 + 2 N MLs, *N* = 4^20^. **c–e** TEM images of **c** 4-ML core, **d** core/crown, and **e** C/C@GS CQWs. **f** High-resolution TEM image of C/C@GS CQWs for 4 + 2 N MLs, *N* = 4. The thickness equals ~4.25 nm, which corresponds to 4 (core) + 2 × 4 (shell) MLs of core/shell heterostructure. **g** Absorbance (black line) and PL (dashed red line) spectra of CdSe/CdS@Cd_1-x_Zn_x_S CQWs for 4 + 2 N MLs, *N* = 4. Inset shows the photoluminescence excitation (PLE) spectra of the sample at different (marked) wavelengths.

For this study, we synthesized CdSe/CdS@Cd_1-x_Zn_x_S C/C@GS CQWs with a carefully controlled different number of Cd_1-x_Zn_x_S-alloyed gradient shell monolayers (MLs), tuned from 1 to 6 MLs, grown on the seed of 4-ML CdSe/CdS core/crown NPLs by using colloidal atomic layer deposition (c-ALD) method^20^. Owing to the tuned gradient-alloyed shell of the NPLs, electron wavefunction feels soft potential confinement in the vertical dimension and is largely relaxed into the shell due to the small conduction band offset, while the hole wavefunction is mostly confined in the core region by virtue of the large valence band offset^20^. This spatial separation between the electron and hole wavefunctions forms quasi-type-II electronic band alignment as previously shown in CdSe/CdS dot/rod heterostructure^7^. The schematic of C/C@GS NPL heterostructure and band-offsets of the heterostructure are given in Fig. 1a, b, respectively. Transmission electron microscopy (TEM) images of the core, core/crown and core/crown@ shell NPLs are shown in Fig. 1c–e, respectively. For the synthesis of C/C@GS hetero-NPLs, we started with 4-ML CdSe cores with lateral dimensions of 13 ± 3 nm, subsequently followed by 4-ML CdS crown (8 ± 1 nm) grown in the lateral direction, and then via using c- ALD method, a gradient-alloyed shell of Cd_1-x_Zn_x_S from 1 to 6 MLs was grown on the core/crown NPLs. Further details of the syntheses procedures are given in Supplementary Note 1. The absorption and photoluminescence (PL) spectra of C/C@GS hetero-NPLs with 4 MLs of Cd_1-x_Zn_x_S shell are depicted in Fig. 1g. PL quantum yield of our NPLs is 85% in solution (hexane). The peaks appearing in the absorption spectrum at 630 and 572 nm are associated with the heavy- and light-hole excitonic transitions, respectively. The PLE spectra of our NPLs taken at different wavelengths are very similar as shown in the inset of Fig. 1g, which indicates a relatively uniform size/shape distribution.

**Fig. 2:**
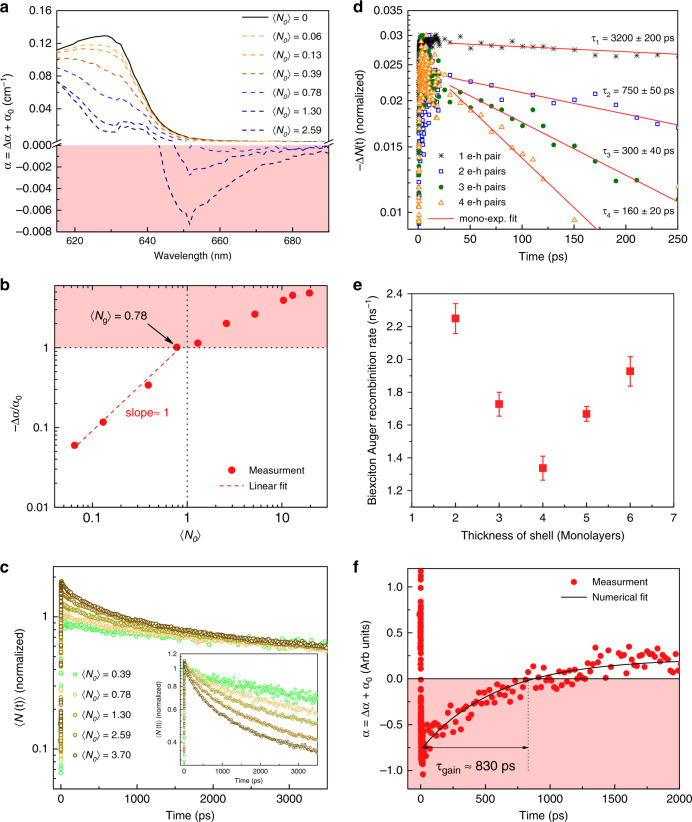
Optical gain characterizations of the synthesized NPLs. **a** Nonlinear absorption (*α*) of 4- ML shell NPLs as a function of 〈*N*_*o*_〉 (from 0 to 2.59, *N*_*o*_ is the number of e–h pairs per NPL), obtained by averaging the recorded TA spectra at early time over 1–5 ps after pumping at 3.1 eV. Red-shaded region exhibits optical gain where *α* < 0. **b** Normalized absorption bleaching (–Δ*α*/*α*_*o*_) as a function of 〈*N*_*o*_〉 at the ASE peak position (*λ*_*ASE*_ = 647 nm) with linear growth at low-pump intensities. The red-shaded region corresponding to optical gain regime (–Δ*α*/*α*_*o*_ > 1) implies a gain threshold of 〈*N*_*g*_〉 = 0.78. **c** Dynamics of pump-dependent average population 〈*N*(*t*)〉 of 4 ML shell at ASE peak position, normalized to long term decay values. The inset shows the normalization to the initial values. **d** Dynamics of 1, 2, 3, and 4 e–h pairs (symbols), obtained from TA spectra of 4 ML shell NPLs at the ASE peak position. The red solid line shows the fit to a single exponential decay. **e** Biexciton Auger recombination rate as a function of the shell thickness. Error bars in this figure come from uncertainty of the fitting parameter (τ_2_) in Fig. 2d and Supplementary Fig. 5. **f** Nonlinear absorption (*α*) as a function of time at the ASE peak position for 〈*N*_*o*_〉 = 1.30 (symbols). Shaded region corresponds to the net optical gain (*α* < 0), indicating the net optical gain lifetime of τ_g_ ≈ 830 s. The solid line is the numerical fitting.

To analyze the optical gain performance of the CdSe/CdS@Cd_1-x_Zn_x_S C/C@GS NPLs with different shell thicknesses, we performed femtosecond transient absorption (TA) measurements of NPLs in solution (hexane) with stirring (see Methods). TA measurements yield spectrally and temporally resolved pump-induced absorption changes (Δ*α*), which were used to calculate the nonlinear absorption spectra (*α* = Δ*α* + *α*_*o*_) in the excited-state, where *α*_*o*_ is the absorption of the unexcited sample. Figure 2a presents *α* for the C/C@GS NPLs with 4 MLs of the shell coating (averaged over 1–5 ps pump-probe delay time) as a function of (*N*_*o*_), where *N*_*o*_ = *f* × *σ* is the number of e–h pairs per NPL. Here, *f* is the fluence and *σ* is the absorption cross-section of the NPLs. The *σ* is calculated based on the method that we discussed in our previous study^21^ (see Supplementary Note 4).

As shown in Fig. 2a, the band-edge absorption is gradually bleaching with increasing (*N*_*o*_) and the transition to optical gain occurs when the absorption bleaching (Δ*α* < 0) is greater than the absorption of the unexcited sample (*α*_*o*_), therefore resulting in a negative net absorption (*α* < 0). To determine the optical gain threshold ((*N*_*g*_)), we depict the normalized absorption bleaching (−Δ*α*/*α*_*o*_) as a function of (*N*_*o*_) at the ASE peak position (λ_ASE_ = 647 nm). Then level of (*N*_*g*_) = 0.78 is quantified from the intersection of the measurement data (symbol) with horizontal dotted line at |Δ*α*/*α*_*o*_| = 1 (Fig. 2b). In addition, the growth of |Δ*α*| follows a nearly linear function (red dashed-line) at initial values of (*N*_*o*_) (Fig. 2b) and hence this feature validates the sub-single exciton level of optical gain in these NPLs. Similar behavior was previously reported for type-II CQDs^16^, where the optical gain occurs in the single-exciton regime.

To investigate the dynamics of the different e–h pair NPL states, we plotted the pump-dependent normalized average e–h pair populations (*N*(*t*)) per NPL at the peak position of stimulated emission (*λ*_ASE_ = 647 nm) (Fig. 2c). Here $N(t) = \mathop {\sum}\nolimits_N {A_Ne^{ - t/\tau _N}}$, where *A*_*N*_ is the time-independent coefficient and *τ*_*N*_ is the lifetime of *N*-pair NPL state. Then, to analyze the multi-excitonic behavior of NPLs (*N* ≥ 2), we employed a simple subtractive process to derive the single exponential decay dynamics as previously used for CQDs^22^ (See Supplementary Note 5 for details). The extracted single exponential dynamics are shown in Fig. 2d, where *τ*_*N*_ corresponds to the lifetime of *N* e–h pair NPL state. As seen in Fig. 2d, by increasing the number of e–h pairs per NPL, the carrier decay kinetics becomes progressively faster, as expected for Auger recombination. The Auger process rate ($\tau _N^{ - 1}$) of an N-exciton state in CdSe-based CQWs follows $a\bar v(N - 1)NA/A_{NPL}$ for *N* ≥ 2, where *ɑ* is the 2D exciton diameter, A_NPL_ is the lateral area of the NPL, A is the Auger recombination probability per collision, and $\bar v$ is the exciton center of mass average relative velocity^23^. This expression results in *τ*_4_:*τ*_3_:*τ*_2_ = 0.17: 0.33: 1, which is in good agreement with our experimental results *τ*_4_:*τ*_3_:*τ*_2_ = 0.21: 0.40: 1,where the extracted lifetimes for 4, 3, and 2 e–h pairs correspond to 160 ± 20, 300 ± 40, and 750 ± 50 ps, respectively.. Here, the single-exciton radiative lifetime (12,100 ± 150 ps) extracted from our time-resolved PL measurements (Supplementary Fig. 4) is ~16-fold longer than the biexciton Auger lifetime. As expected, in the case of sub-single exciton gain regime, the inherent gain kinetics is characterized by the radiative single-exciton lifetime, which is intrinsically much longer than the Auger decay lifetime.

Moreover, by performing the method as described above, we calculated the biexciton Auger recombination rate for 2, 3, 5, and 6 MLs shell thicknesses. As can be seen in Fig. 2e, the biexciton Auger decay rate exhibits a non-monotonic dependence on to the thickness of the shell. The origin of this behavior is the thickness-dependent oscillations in the overlap of the initial and final wavefunctions of the electron in the second exciton as previously shown in core/shell NPLs^24^. The temporal nonlinear absorption (*α*) in the case of 4 ML thick shell at the ASE peak position for (*N*_*o*_) = 1.30 is presented in Fig. 2f, in which the shaded region (*α* < 0) corresponds to net optical gain. Here the gain lifetime was found to be ~830 ps from the intersection of the experimental data by the line of α = 0. As expected, this value is very close to the biexciton Auger decay lifetime (750 ± 50 ps). It is worth mentioning that Auger recombination is suppressed in our engineered NPLs, while for simple core, core/crown and core/shell NPLs, the Auger decay lifetime was reported to be typically 150–500 ps^17,18,24^. The longer biexciton Auger lifetime in the gradient-alloyed shell NPLs can be attributed to the significant reduction in the strength of the intraband transition as a result of the smooth potential confinement^25^. The fine grading of the confinement potential also contributes to the single-exciton gain regime in our gradient-alloyed shell NPLs by slowing down Auger recombination^15,25,26^. The abrupt interfaces were shown to exhibit higher Auger rates as a result of the larger overlap between the initial and final states of the intraband transition^15,25^.

**Fig. 3:**
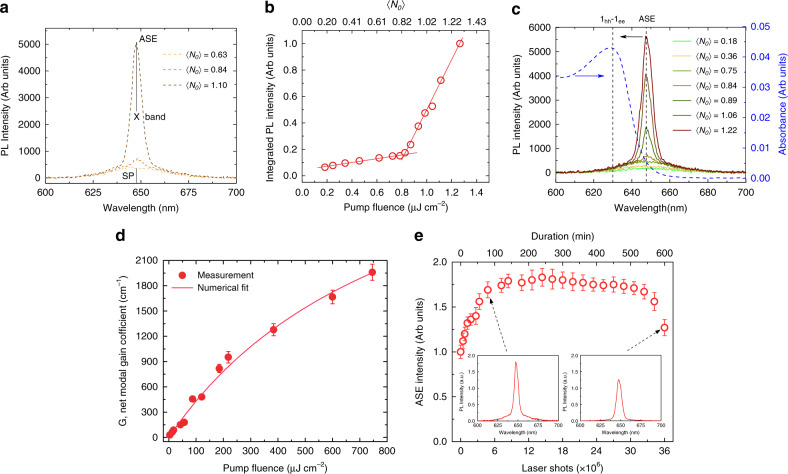
ASE, VSL, and optical gain stability characterizations of the CQWs having four monolayers of shell. **a** Pump-dependent ASE spectra at 〈*N*_*o*_〉 = 0.63, 0.84, and 1.10. The position of spontaneous emission (SP), ASE and X-band are marked by solid black line. **b** Integrated PL intensity of ASE spectra as a function of the pump fluence and the corresponding average number of excitons per NPL (〈*N*_*o*_〉). **c** Spectral analysis of ASE along with absorbance (dashed blue line) of the lowest excitonic energy state with respect to the 〈*N*_*o*_〉. The position of 1_hh_–1_e_ excitonic transition and ASE peak are marked by dark dashed lines. **d** Pump-dependent net peak modal gain coefficient (symbols) at the ASE peak position (*λ*_ASE_ = 647 nm). The solid line is the numerical fitting to the experimental data, indicating a saturation fluence at ~920 μJ cm^−2^. The highest G reaches 1960 ± 100 cm^−1^ at 746 μJ cm^−2^ pump fluence. Error bars in this figure are a result of uncertainty of the fitting parameter in the variable stripe length measurement presented in Supplementary Fig. 7 as exemplary cases. **e** Pump-dependent ASE intensity as a function of the pump laser shot at 〈*N*_*o*_〉 = 1.36. Error bars in this figure comes from the fluctuations in the ASE intensity between different measurements. The light amplification is highly stable in these engineered CQWs even for 9.5 h of continuous excitation.

Next, we investigated the light amplification of CdSe/CdS@Cd_1-x_Zn_x_S C/C@GS CQWs, particularly for 4-MLs shell thickness by performing ASE and variable stripe length (VSL) measurements via stripe geometry excitation of the femtosecond mode-locked laser at 3.1 eV (see Methods). We prepared close-packed thin films of the engineered NPLs via spin-coating technique. The thickness of the ASE and VSL samples is 150 ± 20 nm. Pump-dependent PL spectra of the ASE measurement are shown in Fig. 3a. As can be seen, by increasing the pump fluence, a narrow emission feature develops at a position close to the spontaneous emission (SP) (*λ*_*SP*_ = 646 m). The SP and ASE spectra exhibit full-width-at-half-maxima (FWHM) of ~28 and ~7 nm, respectively. This nonshifted ASE peak (*λ*_*ASE*_ = 647 nm), near the position of the single-exciton band (X-band), exhibits a clear excitation threshold of ~820 nJ cm^−2^, corresponding to (*N*_*o*_) = 0.84 above the threshold, where the integrated intensity of emission follows a super-linear function of the pump fluence. (Fig. 3b) These important observations indicate that net optical gain regime occurs at the sub-single exciton level per particle in our CQWs having smooth confinement potential. Quantitively, the number of excited NPLs in an ensemble sample with different exciton (n) states can be calculated from Poisson distribution: $P\left( n \right) = \left\langle {N_o} \right\rangle ^ne^{ - _{\left\langle {N_o} \right\rangle }}/n!$, where at the ASE threshold (*N*_*o*_) = 0.84, *P*(*n* > 1)≈ 0.21. Then, even in single-exciton gain regime, small but finite loss arises due to Auger process. The effect of Auger recombination is minimized in our gradient alloy core/shell heterostructure owing to the soft potential confinement. This results in remarkably slow Auger recombination (2.5–5 times) in comparison with previous reports of NPLs^17,18,24^.

In addition, the appearance of similar sharp emission is observed with increasing length of the stripe in VSL measurement while keeping the pump intensity fixed (see Methods). Also, the ASE measurements are fully consistent with our TA analysis in the solution form of these NPLs, which indicates an optical gain threshold of (*N*_*g*_) = 0.78. Here we reckon that the successful inactivation of Auger recombination allows to achieve the record lowest ASE threshold (*I*_*ASE*_), which is several orders of magnitude lower than the CdS/ZnSe core/shell type-II CQDs^16^ (*I*_ASE_≈ 2 mJ cm^−2^), CdSe/ZnCdS core/shell type-I CQDs^2^ (*I*_ASE_≈ 90 μJ cm^−2^), and charged CQDs^1^ (*I*_ASE_≈ 6 μJ cm^−2^), in which the concepts of single- or sub-single exciton optical gain were reported. We attribute this superior optical gain performance of our engineered NPLs to their large absorption cross-section at the excitation wavelength (*σ* = 5.06 × 10^−13^ cm^2^ at 400 nm) owing to the core/crown heterostructure along with an efficient exciton funneling^10^.

The lowest excitonic energy state (heavy-hole excitonic transition (1_hh_ − 1_e_)) is shown in Fig. 3c, where the stimulated emission is largely Stokes-shifted with respected to the 1_hh_ − 1_e_ excitonic feature. This major Stokes shift (~17 nm) significantly reduces the reabsorption of the emitted photon, a critical loss mechanism in optical gain, which leads to a remarkably reduced ASE threshold and sub-single exciton optical gain regime. As seen in Fig. 3c, the ASE peak emerges at the red side of the absorption where the emitted photon at the ASE wavelength is absorbed insignificantly. This phenomenon was observed in gain media of colloidal CQDs^2^, epitaxially-grown II–VI quantum well lasers^27^, and organic dyes^28^ as reported previously.

The net modal gain coefficient (G) was also characterized via VSL measurement^10,29^, which we performed at different pump fluences much higher than optical the gain threshold (thus in multi-excitonic regime), by using the integrated intensity of the recorded PL spectra as a function of the stripe length (*l*) and employing the expression *I*_*PL*_(*l*) = *A*/*G*(*e*^*Gl*^ −:1)^10,14^, where A is a constant proportional to the spontaneous emission. The net modal gain coefficient (G) was obtained as presented in Supplementary Note 7. Figure 3d displays G with respect to the pump intensity; for instance, we achieved G = 460 ± 10 cm^−1^ at an excitation fluence of 87 μJ cm^−2^. Here, by increasing the pump fluence, G rises first linearly at the low-pump intensities, then followed by a gradual saturation behavior at elevated excitation fluences. The maximum achieved G is 1960 ± 100 cm^−1^ at 746 μJ cm^−2^. The obtained net modal gain is several-folds higher than the previously reported best performing heterostructures of semiconductor NCs, including core/shell CQDs^2^ (G = 95 ± 10 cm^−1^ at 120 μJ cm^−2^), core/crown NPLs^30^ (G = 929 ± 23 cm^−1^ at 580 μJ cm^−2^), and core/shell NPLs^10^ (G = 600 ± 100 cm^−1^ at 200 μJ cm^−2^). Our group also most recently reported an exceptionally large net modal gain in core NPLs of superior thin film quality as high as 6600 ± 350 cm^−1^ ^14^.

To examine the stability of the light amplification in CdSe/CdS@Cd_1-x_Zn_x_S C/C@GS NPLs, we continuously pumped the ASE sample up to 36 × 10^6^ laser shots (at 1 kHz repetition rate), which corresponds to overall 10 h of continuous excitation. The excitation intensity was ~7 μJ cm^−2^, which is almost 10-fold larger than the ASE threshold. As shown in Fig. 3e, the ASE intensity is stable up to ~9.5 h, which is the record-long stable ASE among all colloidal semiconductor NCs including CQDs^31^ and NPLs^32^, to the best of our knowledge. Therefore, our C/C@GS NPLs offer an effective architecture to substantially improve the optical gain stability issue of colloidal semiconductor NCs as a gain medium owing to ultralow ASE threshold, which is enabled by stimulated emission in the sub-single exciton regime and excellent passivation of the structure (top, bottom, and lateral surfaces) with the crown and shell layers.

**Fig. 4:**
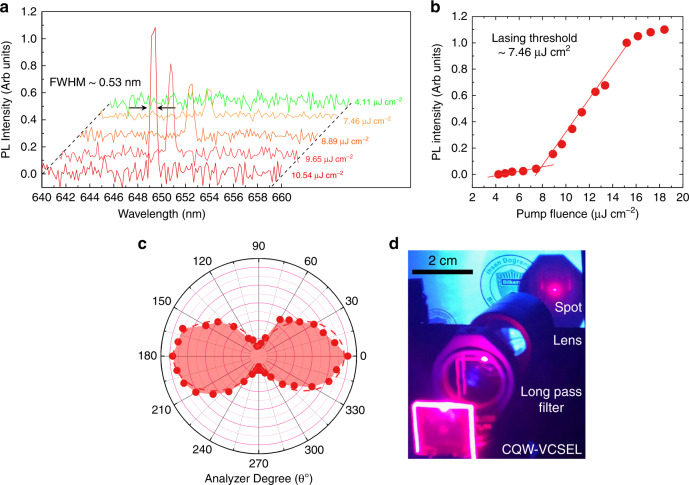
Lasing characterization of CQW–VCSEL. **a** PL spectra of CQWs-VCSEL as a function of the pump fluence. From the linewidth of the laser emission, the quality factor (Q) is calculated as ~1200. **b** Emission intensity versus pump intensity (symbols). The red solid line indicating the lasing threshold of ~7.46 μJ cm^−2^. **c** Normalized emission spectrum of CQWs-VCSEL above the lasing threshold (~12 μJ cm^−2^) as a function of the polarizer angle (*θ*) and the detector at the front of the cavity. The dashed red line is the fitting of cos^2^(*θ*) curve. **d** Photographical image of the CQWs-VCSEL above the lasing threshold indicating a well-defined spatial lasing spot on the screen.

Finally, owing to highly efficient optical gain performance of our engineered CdSe/CdS@Cd_1-x_Zn_x_S C/C@GS CQWs, we incorporated these NPLs (with 4-ML shell thickness) as a gain medium into a vertical-cavity surface-emitting laser (VCSEL). The optical resonator was fabricated by using a pair of distributed Bragg reflectors (DBRs), the reflectance of each of which reaches ~96% at the emission wavelength of the NPLs while keeping lower than ~10% at the excitation wavelength (400 nm). Then, a close-packed solid film of these NPLs was sandwiched between the DBRs (see Methods). The emission spectrum of the CQW–VCSEL is depicted in Fig. 4a at different pump fluences, demonstrating a single-mode lasing with a sharp laser output (FWHM ≈ 0.53 nm) at 649 nm corresponding to a *Q*-factor of ~1200. The lasing output shows a clear excitation threshold of ~7.46 μJ cm^−2^ (Fig. 4b) with a well-defined spatial coherence profile (spot in Fig. 4d). This lasing threshold is the lowest among all previously reported colloidal semiconductor NC-based VCSELs, for which the next lowest reported lasing threshold is ~60 μJ cm^−2^ ^2^. As can be seen in Fig. 4b, the pump-dependent emission displays an S-shaped curve (lasing intensity saturated at the elevated pump fluences > 15.2 μJ cm^−2^), which is one of the characteristic identifications of the lasing action. The factor (*R*) of the polarization can be defined as *R* = (*I*_||_ − *I*_⊥_)/(*I*_||_ + *I*_⊥_)^33^, where *I*_||_ and *I*_⊥_ is the intensity parallel and perpendicular to the optical axis, respectively. Figure 4c shows the lasing emission with respect to the polarizer angle, the dumbbell distribution of the measured data (symbols) indicating a linearly polarized emission of our fabricated laser, which is well fitted by a quadratic cosine function (cos^2^(*θ*)) where *R* = 0.82 (see Supplementary Fig. 9).

## Discussion

The conducted study presents a demonstration of light amplification in sub-single exciton gain regime of CQWs having smooth confinement potential, which avoids the complications associated with fast nonradiative multi-excitonic Auger process. This was confirmed by systematic nonlinear absorption analyses and linear dependence of normalized absorption changes below gain. This sub-single exciton ((*N*_*g*_)≈ 0.84) optical gain, accompanied with extremely large absorption cross-section, in CdSe/CdS@CdZnS core/crown@gradient-alloyed shell NPLs enables record low gain threshold among all colloidal semiconductor nanocrystals. The ultrastable gain performance of these engineered NPLs addresses the stability problem of colloidal nanocrystals as a gain medium. Finally, the developed CQW–VCSEL exhibit a record low threshold of single-mode lasing. The realization of sub-single exciton regime optical amplification along with ultrastable characteristics in these CQWs may enable lasing under continuous-wave excitation, possibly leading to electrically driven CQW lasers.

## Methods

### Ultrafast transient absorption measurements

TA spectroscopy was performed using a Helios^TM^ setup (Ultrafast Systems LLC) and in transmission mode with chirp-correction. The white light continuum probe beam (in the range of 400–800 nm) was generated from a 3 mm sapphire crystal using 800 nm pulse from the regenerative amplifier. The 400-nm pump laser pulses were generated from a 1 kHz regenerative amplifier (Coherent Libra^TM^). The beam from the regenerative amplifier has a center wavelength at 800 nm and a pulse width of around 150 fs and is seeded by a mode-locked Ti-sapphire oscillator (Coherent Vitesse, 80 MHz). The 400-nm pump laser was obtained by frequency doubling the 800-nm fundamental regenerative amplifier output using a BBO crystal. The pump beam spot size was ~0.5 mm. The probe beam passing through the sample in cuvette was collected using a detector for UV–Vis (CMOS sensor). All measurements were performed at room temperature in solution (hexane) with stirring.

### ASE measurements

As the pump source, we used a femtosecond mode-locked Ti: sapphire regenerative amplifier (Spectra Physics, Spitfire Pro) having a 120 fs pulse width and a 1 kHz repetition rate, operating at the frequency-doubled output (400 nm by using a BBO crystal). A variable neutral density filter was employed to change the pump fluence on the sample. For the stripe geometry excitation, a cylindrical lens with a 10 cm focal length was used. Then, the photoluminescence signal was collected at the end of the stripe by a fiber coupled to a spectrometer (Maya 2000 Pro), where the collection was perpendicular to the excitation. For the ASE samples, we fabricated a thin film of CdSe/CdS@Cd_1-x_Zn_x_S C/C@GS CQWs by using a highly concentrated solution (40–50 mg mL^−1^ of the CQWs in hexane) via spin-coating (at 1000 rpm for 1 min) on quartz substrates.

### VSL measurements

Sample preparation was very similar to ASE samples. The optical setup was slightly modified, where an adjustable slit was used to vary the length of stripe on the sample and also to minimize the effect of light diffraction, the sample was placed as close as possible to the slit (~4–5 mm). To ensure 1D amplifier assumption and to maximize the ASE intensity from the edge of sample, the length of the excitation was adjusted to be ~120 μm. The PL signal was collected at the edge of the sample.

### Fabrication of the CQW–VCSEL

To fabricate highly reflective DBRs for CQW-VCSELs, eight pairs of SiO_2_/TiO_2_ (with the optical thickness of silica and titania chosen as quarter-wavelength emission of CQWs) were deposited on quartz substrate via sputtering. A narrow stripe of 120-μm thick Kapton tape was placed at the edge of one of the DBRs. On the other side, using an epoxy, two DBRs were stick to each other by applying slight pressure, and then the tape was removed. This creates a finite wedge-shaped structure between two DBRs, which provides a built-in thickness variation in the resulting vertical cavity. Finally, a highly concentrated (50–60 mg mL^−1^) CQW/hexane was poured and dried between two DBRs, together with which formed a cavity length variable Fabry–Perot resonator.

### Lasing measurements

The pump source was the same that as we used in ASE measurement. For the spot-excitation geometry, a spherical lens with a 2.5 cm focal length was used. Then, the photoluminescence signal of the laser device was collected at the back of the sample by a fiber coupled to a spectrometer (Avantes, AVASPEC-ULS3648TEC) with a spectral resolution of 0.16 nm, where the collection direction was parallel to the excitation.

### Reporting summary

Further information on research design is available in the Nature Research Reporting Summary linked to this article.

## Supplementary information

Supplementary Information

Reporting Summary

## Data Availability

The raw experimental and numerical data used in this study are available from the corresponding author upon reasonable request. The source data underlying Figs. 1g, 2a–f, 3a–e, and 4a-c and Supplementary Figs. 2, 3, 4, 5a–d, 6, 7a–d, 8a, and 9 are provided as a Source Data file.

## Source data

Source data
