# Supplementary material for: Sub-single exciton optical gain threshold in colloidal semiconductor quantum wells with gradient alloy shelling
Source: Nat Commun. 2020 Jul 3;11:3305. doi: 10.1038/s41467-020-17032-8 (PMC7335098; doi:10.1038/s41467-020-17032-8)
Supplement: Supplementary file 1 — Supplementary Information [file 41467_2020_17032_MOESM1_ESM.pdf]

Supplementary Information

Sub-single exciton optical gain threshold in colloidal semiconductor quantum wells with gradient alloy shelling

Taghipour et al.

Supplementary Note 1: Synthesis of core/crown@gradient-alloyed shell CdSe/CdS_xZn_xS colloidal quantum wells

Preparation of cadmium myristate: Cadmium myristate was prepared according to a previously reported recipe[1]. 1.23 g of cadmium nitrate tetrahydrate was dissolved in 40 mL of methanol and 3.13 g of sodium myristate was dissolved in 250 mL of methanol. When both the powders were completely dissolved, the solutions were mixed and stirred vigorously for 1 h. Cadmium myristate was formed as a precipitate, which was then removed by centrifugation and washed by redispersing in methanol to remove any unreacted and/or excess precursors. After repeating the washing step for at least three times, the precipitated part was completely dried under vacuum overnight.

Synthesis of the 4ML thick CdSe core NPLs: We followed the recipe reported previously with slight modifications[1]. 170 mg of cadmium myristate, 12 mg of Se and 15 mL of ODE were loaded into a three-neck flask. After degassing the mixture for 1 h at room temperature, the solution was heated to 240 °C under argon atmosphere. When the solution turns bright orange (generally around 190-200 °C), 80 mg of cadmium acetate dihydrate was swiftly added to the reaction solution. After reaching 240 °C, the solution was cooled to room temperature and 0.5 mL of OA was injected. CdSe NPLs were precipitated by adding acetone and dispersed in hexane. Size-selective precipitation using centrifugation at different speeds was used if any additional sizes of NPLs were formed.

Preparation of anisotropic growth solution for CdS crown: A previously reported procedure was followed with slight modifications[1]. For the preparation of cadmium precursor, 480 mg of cadmium acetate dihydrate, 340 µL of OA, and 2 mL of ODE were loaded in a beaker. The

solution was sonicated for 30 min at room temperature. Then, it was heated to 160 °C in ambient atmosphere under continuous stirring and alternating sonication until the formation of whitish color homogeneous gel. After the cadmium precursor was prepared, it was mixed with 3 mL of 0.1 M S-ODE stock solution and used for the CdS crown coating.

Synthesis of CdSe/CdS core/crown NPLs: A typical core-seeded synthesis method reported previously was used with slight modifications[1]. A portion of the 4 ML core synthesis in hexane and 15 mL of ODE were loaded in a three-neck flask. The solution was degassed at 100 °C for the complete removal of hexane. Then, the solution was heated to 240 °C under argon flow and a certain amount of anisotropic growth mixture for CdS crown was injected at the rate of 12 mL/h. After obtaining the desired crown size by adjusting the injection amount, the resulting mixture was further annealed at 240 °C for 5 min. After that, the solution was cooled down to room temperature and the core/crown NPLs were precipitated using ethanol. The NPLs were cleaned three times with ethanol and methanol to remove any traces of unreacted precursors, which was crucial for the shell growth step using c-ALD. Lastly, they were then dispersed in hexane to be used for the shell deposition.

Synthesis of CdSe/CdS@$Cd_{1-x}Zn_xS$ core/crown@shell NPLs: We used a modified procedure of our c-ALD recipe reported previously[2, 3]. 1 mL of core/crown NPL seeds in hexane (having first absorption peak at 514 nm) were kept for use such that 100 μL of these NPLs dissolved in ~3 mL hexane had an optical density of ~2 at 370 nm. For cation precursors we used 0.4 M cadmium nitrate tetrahydrate (Cd-nitrate) and 0.4 M zinc nitrate hexahydrate (Zn-nitrate) solutions in NMF. For sulfur precursor we used 40-48 wt% solution of ammonium sulfide in water. Specifically, for the first sulfur shell growth, we added 40 μL of ammonium sulfide in 4 mL NMF and under vigorous stirring added 1 mL of CdSe/CdS core/crown seeds, which we had

prepared separately. After 2 minutes of stirring when all the NPLs had entered the NMF phase from hexane, the reaction was stopped by quickly adding acetonitrile and excess toluene to precipitate the NPLs via centrifugation. This cleaning step was repeated once more by redispersing the NPLs in NMF and precipitating them using acetonitrile and excess toluene to remove any remaining sulfur precursor. Finally, the NPLs were dispersed in 4 mL of NMF for the next cation deposition step. Next, we added 1 mL of solution having a mixture of *X*% Cd-nitrate and (100-*X*)% Zn-nitrate in NMF by volume for the cation step. The *X* (volume percentage of Cd precursor) value was varied as 50, 10, 5, 2, 1 and 1 from first to the sixth $Cd_{1-x}Zn_xS$ shell layers. The reaction was allowed to continue by stirring for at least 45 min in ambient atmosphere and under room light, after which the NPLs were precipitated out by adding acetonitrile and excess toluene for centrifugation. The cleaning step similar to the previous one was repeated once more to remove all excess precursors. The growth cycle of sulfur and cation precursors were further continued (now only in NMF) to increase the number of shells as required while gradually increasing the Zn content with each cycle as indicated above. Each cycle added 1 monolayer of $Cd_{1-x}Zn_xS$ shell on top of the previous. At the end of each cycle a small amount of the NPLs were precipitated from NMF and dispersed in hexane by adding oleylamine for further use.

Supplementary Note 2: Quantum mechanical calculation of $CdSe/CdS@Cd_{1-x}Zn_xS$ core/crown@gradient-alloyed shell colloidal quantum wells

Calculations of electron and hole wavefunctions: To calculate the electron and hole wavefunctions for the first excited state, we solved time–independent Schrödinger equation (TISE) for the potential profile given in Supplementary Figure 1:

$$\left[\frac{\hbar^2 d^2}{2mdz^2} + V(z)\right]\Psi(z) = E_z\Psi(z) \qquad (1)$$

where m_z is the effective mass of electron (hole), $V(z)$ is the potential arising from conduction (valence) band offsets, and $\Psi(z)$ is the wave function of electron (hole). We assumed the even parity for the symmetry of first excited states and solved the TISE for $z > 0$. Then, because of the symmetry, we reflected the obtained solution to $z < 0$.

To solve the problem, we divided the right half of the potential well into five regions, as indicated in Supplementary Figure 1, and write the general solutions for each region:

Region I, by assuming even symmetry:

$$\Psi(z) = Acos(kz) \qquad (2)$$

where $k = \sqrt{\frac{2m_1E}{\hbar^2}}$.

For Regions II, III, IV and V:

$$\Psi(z) = C_{2,3,4,5}e^{K_{2,3,4,5}z} + D_{2,3,4,5}e^{-K_{2,3,4,5}z} \qquad (3)$$

where $K_{2,3,4,5} = \sqrt{\frac{2m_{2,3,4,5}(V_{1,2,3,4}-E)}{\hbar^2}}$.

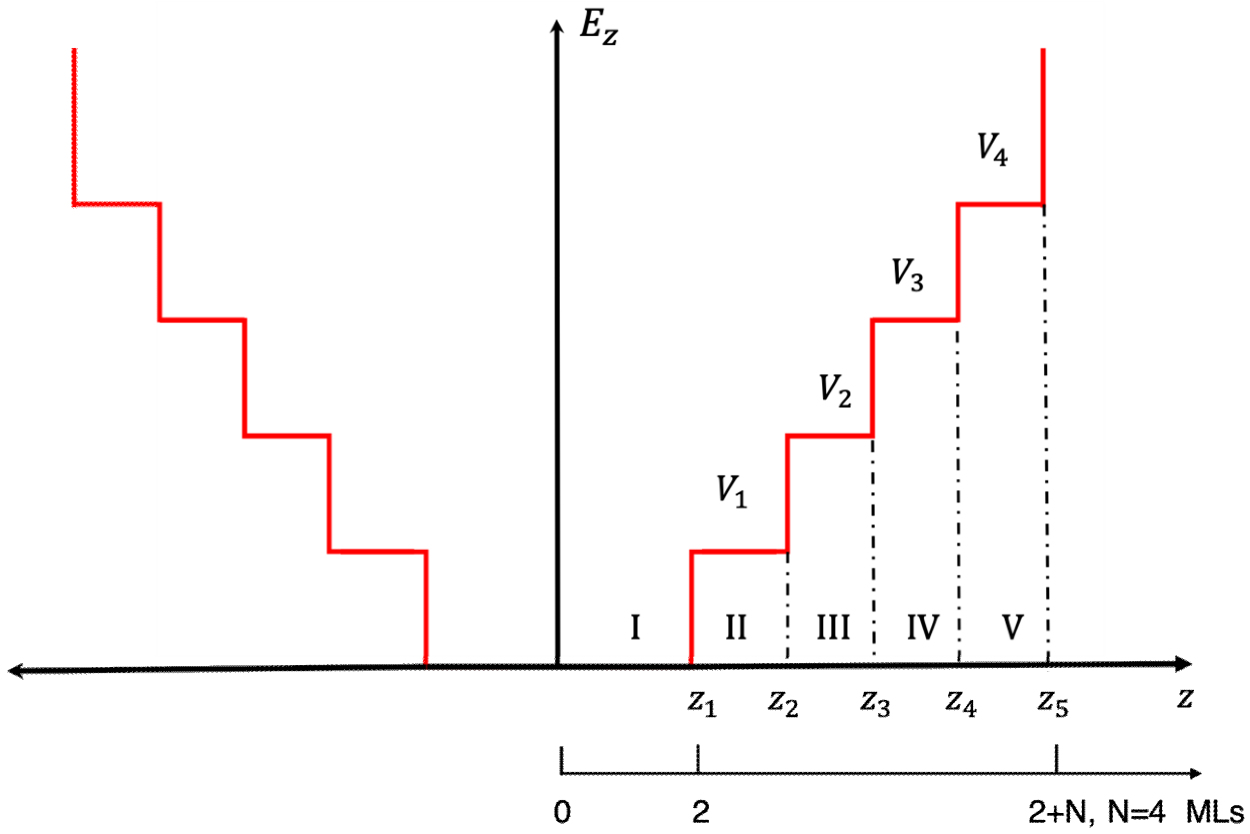

Supplementary Figure 1. Potential diagram of CdSe core coated with N=4-MLs $Cd_{1-x}Zn_xS$ gradient alloyed-shell. Here, x represents the amount of Zn in the shell region, where x is 0.02%, 13.5%, 24.6% and 53.2% for 1st, 2nd, 3rd and 4th MLs of the shell coating, respectively. V_1 is the potential barrier offset between 4-ML CdSe core and CdS shell. V_{2-4} are the potential barrier offsets for the electron (or hole) wavefunctions between CdSe core and $Cd_{1-x}Zn_xS$ shell for different amount of x.

Then, we solved the equations for electron (hole) by using the following boundary conditions:

$$\Psi\left(z_{1,2,3,4}^{-}\right) = \Psi\left(z_{1,2,3,4}^{+}\right) \qquad (4)$$

$$\frac{d\Psi(z)}{dz}\Big|_{z_{1,2,3,4}^{-}} = \frac{d\Psi(z)}{dz}\Big|_{z_{1,2,3,4}^{+}} \qquad (5)$$

$$\Psi(z_5) = 0 \qquad (6)$$

To do so, “A” assumed to be 1 for finding the general solution, and then it was obtained by applying the normalization condition:

$$\int_{-\infty}^{+\infty} |\Psi|^2 dz = 1 \qquad (7)$$

In our calculations, for the value of effective masses and band offsets, we used the data that are given in Supplementary References 3.

Solutions;

At the interface between Region I-II ($\mathbf{z = z_1}$):

From Supplementary Equations 2 and 3 and by applying the boundary conditions of Supplementary Equations 4 and 5:

$$C_2 = \frac{K_2 \cos(kz_1) - k \sin(kz_1)}{2K_2 e^{K_2 z_1}} \text{ and } D_2 = \frac{K_2 \cos(kz_1) + k \sin(kz_1)}{2K_2 e^{-K_2 z_1}}$$

In region II-III, III-IV and IV-V interfaces ($\mathbf{z = z_{2,3,4}}$)

From Supplementary Equation 3 and applying the boundary conditions of Supplementary Equation 4 and 5:

$$C_{3,4,5} = \frac{C_{2,3,4}\left(K_{3,4,5} + K_{2,3,4}\right) e^{K_{2,3,4,5} z_{2,3,4}} + D_{2,3,4}\left(K_{3,4,5} - K_{2,3,4}\right) e^{-K_{2,3,4,5} z_{2,3,4}}}{2K_{3,4,5} e^{K_{3,4,5} z_{2,3,4}}} \qquad (8)$$

and

$$D_{3,4,5} = \frac{C_{2,3,4}\left(K_{3,4,5} - K_{2,3,4}\right) e^{K_{2,3,4,5} z_{2,3,4}} + D_{2,3,4}\left(K_{3,4,5} + K_{2,3,4}\right) e^{-K_{2,3,4,5} z_{2,3,4}}}{2K_{3,4,5} e^{-K_{3,4,5} z_{2,3,4}}} \qquad (9)$$

Finally, by applying the boundary condition Supplementary Equation 6, we calculated the first excited energy state (E) and corresponding electron (hole) wavefunctions, $\Psi(z)$. The $[\Psi(z)]^2$ is depicted as function of z in Supplementary Figure 2.

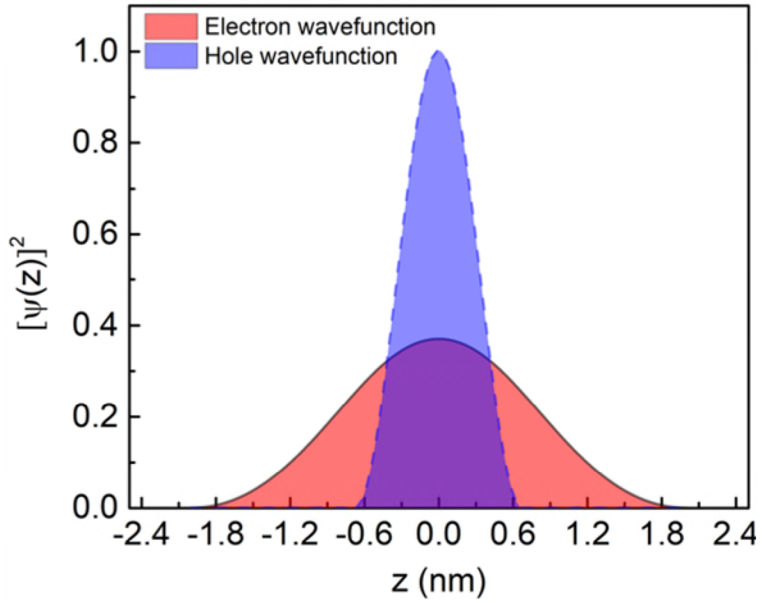

Supplementary Figure 2. Distribution of electron and hole wavefunctions. Calculated the square of electron (red) and hole (blue) wavefunctions for CdSe/CdS/$Cd_{1-x}Zn_xS$ core/crown@gradient alloyed-shell of 4+2N MLs, with N=4 MLs (shell thickness).

As can be seen in Supplementary Figure 2, in our engineered heterostructure of CQWs, the hole wavefunction is mostly confined in the core region due to large band offset (0.52 eV) and heavy effective mass (0.89 m_o) from -0.6 to $+0.6$ nm in Figure S2. However, the electron wavefunction relaxes to the shell region, because of the weak potential barrier of the conduction band (~0.1eV) and light electron effective mass (0.13 m_o). Consequently, this partial separation of electron and hole wavefunctions results in quasi-type-II band alignment.

Supplementary Note 3: PL spectrum and Photoluminescence decay curve of CQWs

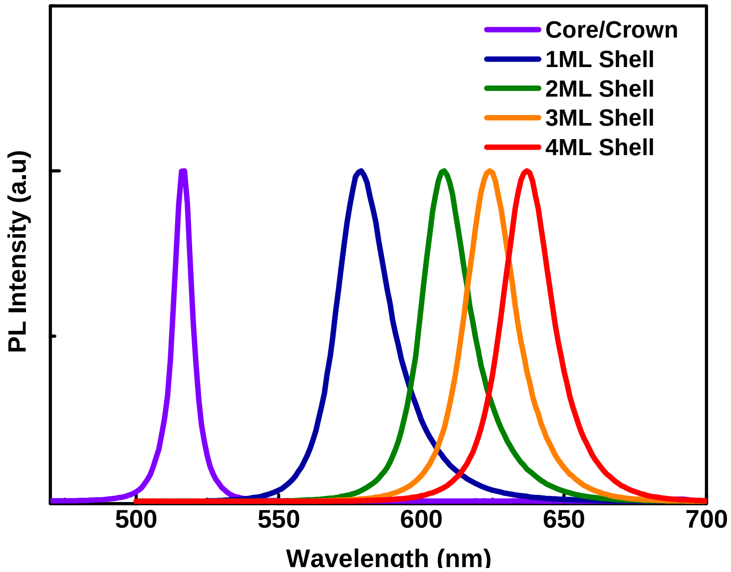

Supplementary Figure 3. PL spectra of $CdSe/CdS/Cd_{1-x}Zn_xS$ core/crown@gradient alloyed-shell CQWs at different shell thicknesses.

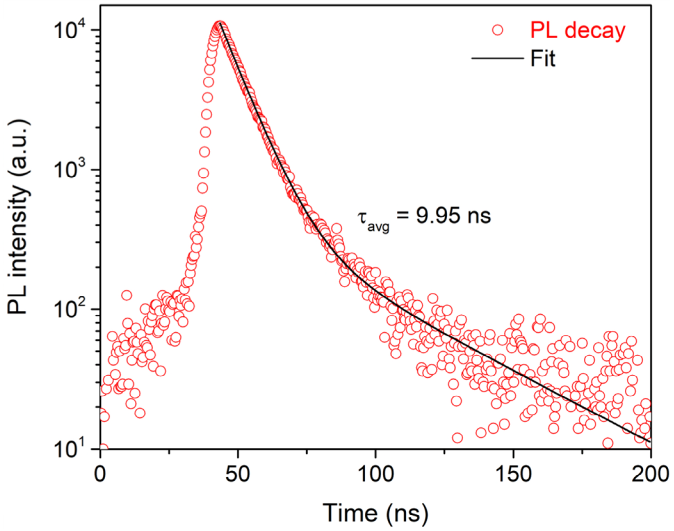

Supplementary Figure 4. Photoluminescence decay curve of $CdSe/CdS@Cd_{1-x}Zn_xS$ core/crown@alloyed-shell CQWs having 4 MLs of shell collected at single-exciton emission peak. The photoluminescence decay curve was fitted with a biexponential decay function. The lifetime components obtained from the fitting are 4.76 ns and 12.10 ns and the resulting amplitude-averaged PL lifetime is 9.95 ns.

Supplementary Note 4: Calculation of average number of excitons per CQW

Absorption cross-section calculations of NPLs: First, inductively coupled plasma-mass spectroscopy (ICP-MS) measurement was used to calculate the concentration of NPLs. For the ICP-MS measurements, a solution of 1 mL of NPL sample was carefully dried. Then, the residual was dissolved in 5 mL nitric acid solution. The processed sample was placed into a volumetric flask for the ICP-MS measurement. The cadmium and zinc molar concentrations were obtained using an Agilent 725 ICP-MS system.

The size of the CdSe NPLs were obtained from transmission electron microscopy (TEM) images. NPL concentration (C_{NPL}) was calculated using $C_{NPL}= C\times(Cd+Zn)\times V_{unit}/(4V_{NPL})$, where V_{unit} is the volume of the CdSe unit cell, V_{NPL} is the physical volume of CdSe NPL and C×(Cd+Zn) is the total concentration of the Cd and Zn[4]. Herein, we make an assumption that the density of the NPLs is the same as its bulk material. After the calculation of NPL concentration, per-particle absorption cross-section (σ) of CdSe/CdS@CdZnS core/crown@shell NPLs was calculated by employing the equation of $\sigma =2303A/(C_{NPL}\times N_A\times L)$, where A is the absorbance, N_A is the Avogadro's number, and L is the optical path length.

Calculation of Zn concentrations in the CdSe/CdS@CdZnS core/crown/shell NPLs: Zn concentrations in the NPLs were acquired by using ICP-MS measurements. First, Zn concentration of all samples was calculated through ICP-MS measurements. Then, the Zn concentration at each shell layer was calculated by knowing the relative Zn concentration of each sample acquired through consecutive c-ALD procedures and considering the fact that each monolayer has the same volume as a result of their 2D planar geometry.

The calculated Zn concentration in each layer of shells is given in Supplementary Table 1.

Supplementary Table 1. Calculated the Zn concentration of in CdSe/CdS/CdS@$Cd_{1-x}Zn_xS$ core/crown@gradient-alloyed shell NPLs.

Shell number	Zn concentration (%)
1	0.02
2	13.50
3	24.60
4	53.20
5	81.60
6	86.50

Supplementary Note 5: Biexciton Auger recombination lifetime calculation.

By using the method that we mentioned in the main text, we employed a simple subtractive process to derive single exponential decay dynamics as previously used for CQDs[5]. The biexciton Auger recombination rate as a function of the shell layers is shown in Figure 2e.

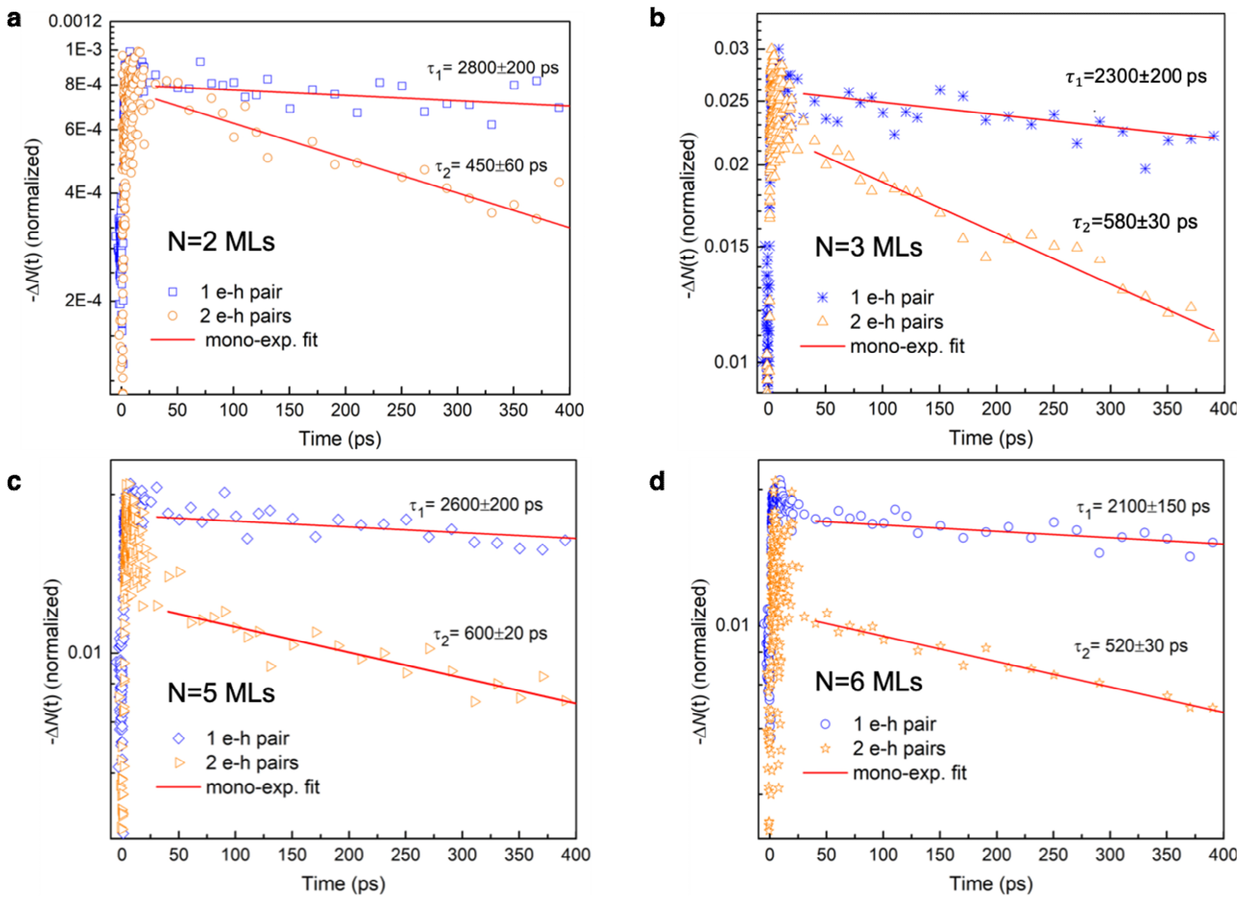

Supplementary Figure 5. Calculated biexciton Auger recombination lifetime for CdSe/CdS@$Cd_{1-x}Zn_xS$ core/crown/gradient alloyed-shell CQWs with different shell thicknesses**. a,** N=2, **b,** N=3, **c,** N=5 and **d,** N=6 MLs.

Supplementarty Note 6: Amplified spontaneous emission measurement

We examined amplified spontaneous emission (ASE) of different samples in the same setup. To do so, we prepared the thin films of CdSe/CdS@$Cd_{1-x}Zn_xS$ C/C@GS CQWs, by applying the similar recipe for all samples.

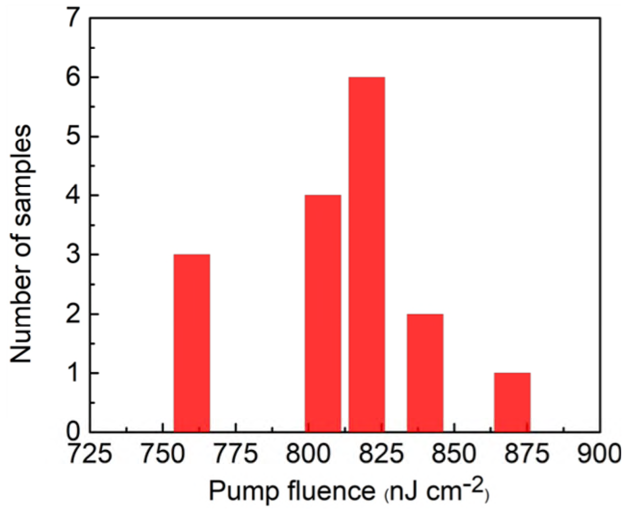

Supplementary Figure 6. Amplified spontaneous emission characterizations. Histogram of ASE thresholds for different thin films of CdSe/CdS@$Cd_{1-x}Zn_xS$ CQWs for 4-ML shell.

Supplementary Note 7: Variable stripe length measurement

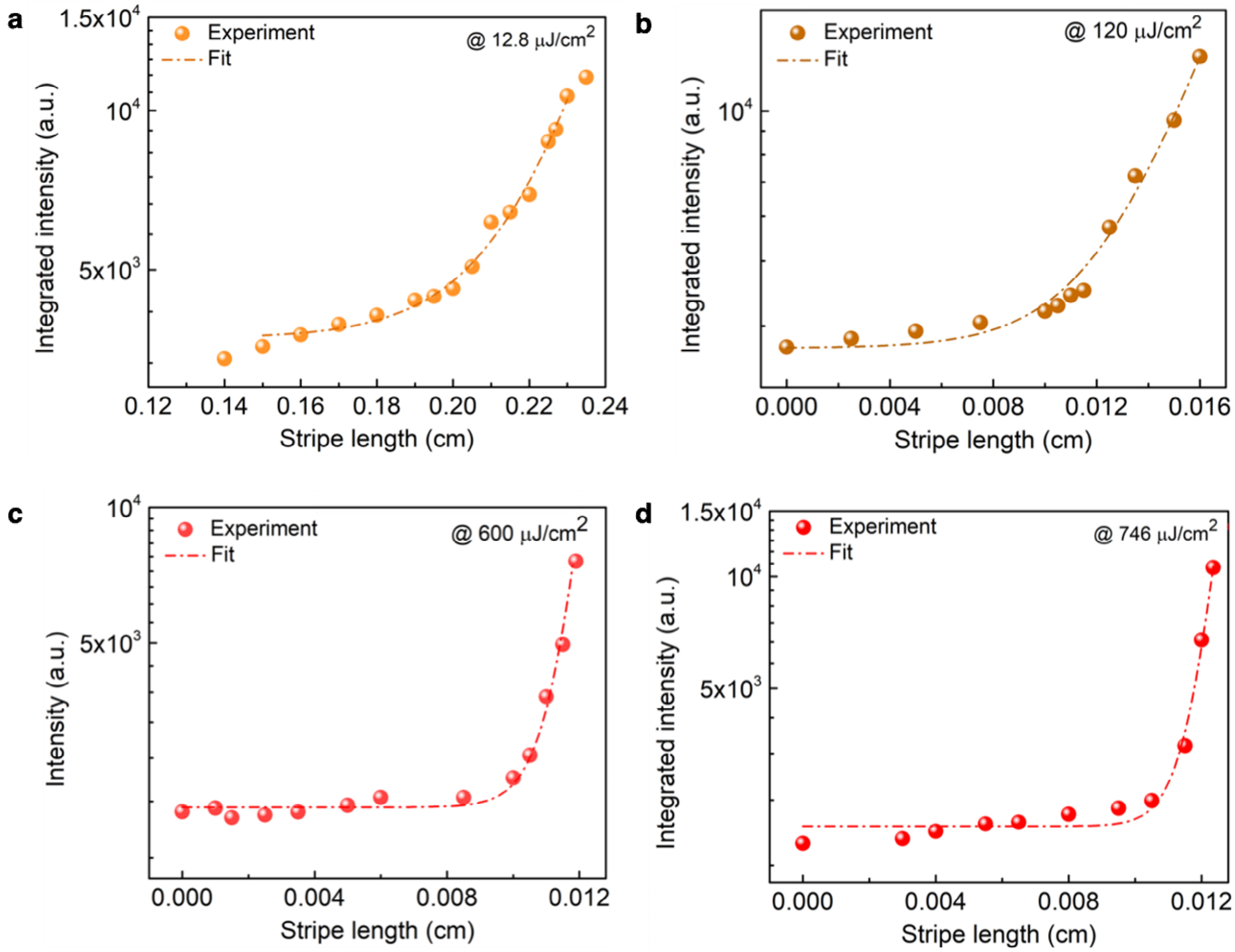

Supplementary Figure 7. Variable stripe length measurements of CdSe/CdS@$Cd_{1-x}Zn_xS$ CQWs for 4-ML shell at different pump fluences. **a**, 12.8, **b**, 120, **c**, 600, and **d**, 746 μJ cm^{-2}. The obtained modal gain coefficients are 60 cm^{-1} for 12.80 μJ cm^{-2} (**a**), 480 cm^{-1} for 120 μJ cm^{-2} (**b**), 1650 cm^{-1} for 600 μJ cm^{-2} (**c**) and 1960 cm^{-1} for 746 μJ cm^{-2} (**d**).

Supplementary Note 8: Colloidal quantum well- vertical- cavity surface-emitting laser.

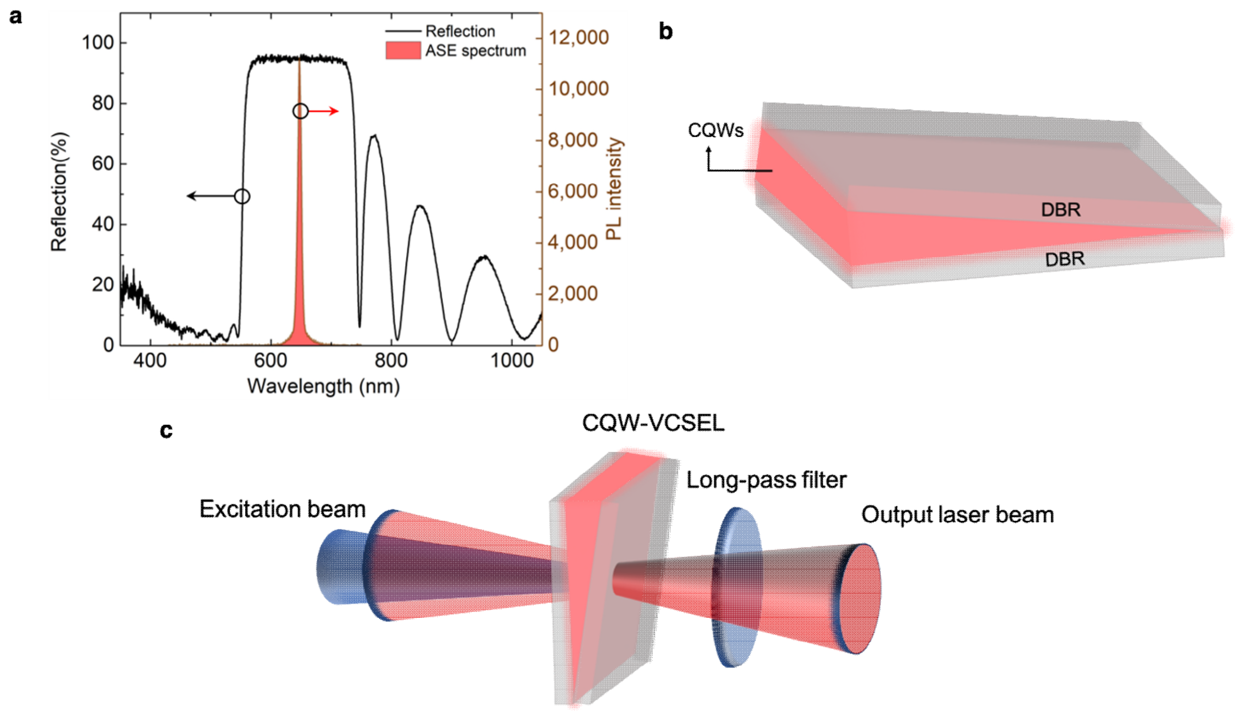

Supplementary Figure 8. CQW-VCSEL. a, Reflection spectra of the DBRs and ASE spectrum of CdSe/CdS@$Cd_{1-x}Zn_xS$ CQWs with 4-ML shell. b, Schematic illustration of wedge CQW-VCSEL consists of two DBRs having a reflection of ~96% where a close-packed solid film of CQWs is sandwiched between them. c, Schematic of vertically-pumped CQW-VCSEL wedge which provides a variable cavity length. To eliminate any residual excitation beam, we used a long-pass filter. The output laser beam was collected with a spectrometer.

Polarization measurements of output laser beam: To characterize the polarization state of the laser emission, a linear polarizer was placed between the cavity output and spectrometer. By varying the angle of the polarizer, the signal was collected at the spectrometer. The luminescence intensity of the cavity is depicted as function of the detection angle in Figure S6, where the polarization factor was found to be 0.82.

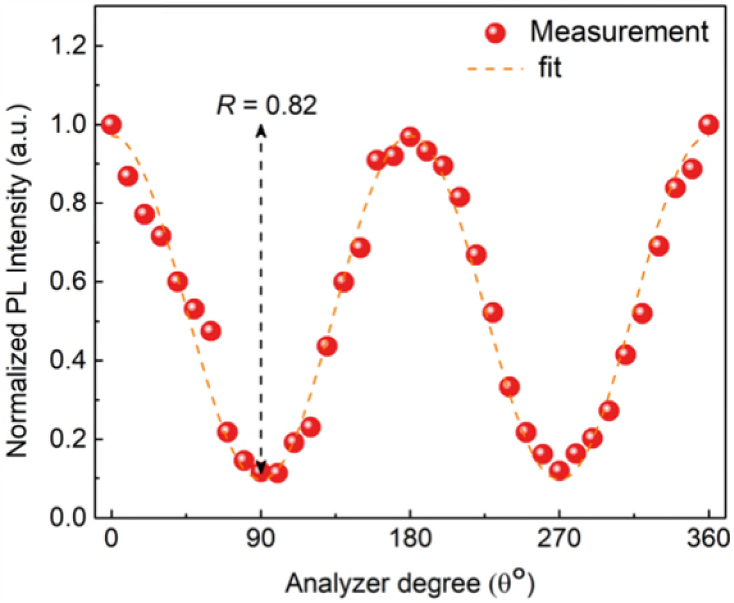

Supplementary Figure 9. PL intensity of the collected signal *versus* detection angle. The polarization factor was calculated using experssion of $R = (I_{\parallel} - I_{\perp})/(I_{\parallel} + I_{\perp})$, which leads to 0.82. The experimental data is well fit with a $\cos^2$ function.

Supplementary References

1. Tessier, M. I. D., Spinicelli, P., Dupont, D., Patriarche, G., Ithurria, S. & Dubertret, B. Efficient exciton concentrators built from colloidal core/crown CdSe/CdS semiconductor nanoplatelets. *Nano Lett.*, 2014, **14**, 207-213.
2. Kelestemur, Y. *et al.* Platelet-in-Box colloidal quantum wells: CdSe/CdS@CdS core/crown@shell heteronanoplatelets. *Adv. Funct. Mater.* **26,** 3570–3579 (2016).
3. Shendre, S. *et al.* Ultrahigh-efficiency aqueous flat nanocrystals of $CdSe/CdS@Cd_{1-x}Zn_xS$ colloidal core/crown@alloyed-shell quantum wells. *Nanoscale* **11,** 301–310 (2019).
4. Yeltik, A. *et al.* Experimental determination of the absorption cross-section and molar extinction coefficient of colloidal CdSe nanoplatelets. *J. Phys. Chem. C* **119,** 26768–26775 (2015).
5. Klimov, V. I., Mikhailovsky, A. A., Mcbranch, D. W., Leatherdale, C. A. & Bawendi, M. G. Quantization of multiparticle Auger rates in semiconductor quantum dots. *Science,* **287,** 1011–1013 (2000).
