# Supplementary material for: Sub-single exciton optical gain threshold in colloidal semiconductor quantum wells with gradient alloy shelling
Source: Nat Commun. 2020 Jul 3;11:3305. doi: 10.1038/s41467-020-17032-8 (PMC7335098; doi:10.1038/s41467-020-17032-8)
Supplement: Supplementary file 2 — Reporting Summary [file 41467_2020_17032_MOESM2_ESM.pdf]

nature research

Corresponding author(s): Professor Hilmi Volkan Demir
volkan@stanfordalumni.org

Lasing Reporting Summary

Nature Research wishes to improve the reproducibility of the work that we publish. This form is intended for publication with all accepted papers reporting claims of lasing and provides structure for consistency and transparency in reporting. Some list items might not apply to an individual manuscript, but all fields must be completed for clarity.

For further information on Nature Research policies, including our data availability policy, see Authors & Referees.

ü Experimental design

Please check: are the following details reported in the manuscript?

1. Threshold

Plots of device output power versus pump power over a wide range of values indicating a clear threshold

- [x] Yes
- [ ] No

- We plotted PL intensity-pump fluence curve lasing actions in Figure 4b.
- A clear lasing threshold was shown in experiment (7.46 uJ/cm2 for lasing), where significant changes in slopes are evident.

2. Linewidth narrowing

Plots of spectral power density for the emission at pump powers below, around, and above the lasing threshold, indicating a clear linewidth narrowing at threshold

- [x] Yes
- [ ] No

-The emission spectra were plotted below and above the lasing threshold in Figure 4a.
- Below the lasing threshold (7.46 uJ/cm2), there is no sharp peak in the emission spectrum (linewidth of spontaneous emission is 28 nm, given in Figure 3a), where at the lasing threshold a narrow peak with FWHM of 0.53 nm (corresponding to a Q-factor of 1,200) appears in the emission spectrum. Also, the appearance of this narrow peak corresponds to the slope change of PL intensity-pump fluence (Figure 4b).

Resolution of the spectrometer used to make spectral measurements

- [x] Yes
- [ ] No

- The spectral resolution of the spectrometer (Avantes, AVASPEC-ULS3648TEC) is 0.16 nm, which is given in Method section.

3. Coherent emission

Measurements of the coherence and/or polarization of the emission

- [x] Yes
- [ ] No

- The polarization measurement of laser device was given in Figure 4c and Supplementary Figure 9.
- The dumbbell distribution of the measured data in Figure 4c, indicates a linearly polarized emission of the laser, which is well fitted by a quadratic cosine function.
- The factor of the polarization (R) is 0.82 (Supplementary Figure 9).

4. Beam spatial profile

Image and/or measurement of the spatial shape and profile of the emission, showing a well-defined beam above threshold

- [x] Yes
- [ ] No

- A photographical image of the CQWs-VCSEL above the lasing threshold was presented in Figure 4d, indicating a well-defined spatial lasing spot on the screen.

5. Operating conditions

Description of the laser and pumping conditions
Continuous-wave, pulsed, temperature of operation

- [x] Yes
- [ ] No

- We stated clearly the pumping condition in Method section. A femtosecond mode-locked Ti: sapphire regenerative amplifier (Spectra Physics, Spitfire Pro) having a 120 fs pulse width and a 1 kHz repetition rate, operating at the frequency-doubled output (400 nm by using a BBO crystal) was used for pumping

Threshold values provided as density values (e.g. W cm^{-2} or J cm^{-2}) taking into account the area of the device

- [x] Yes
- [ ] No

- Pump fluence levels are all provided in the units of uJ/cm2, including the lasing threshold which is of 7.46 uJ/cm2 (Figure 4b).

6. Alternative explanations

Reasoning as to why alternative explanations have been ruled out as responsible for the emission characteristics
e.g. amplified spontaneous, directional scattering; modification of fluorescence spectrum by the cavity

- [x] Yes
- [ ] No

- We performed amplified spontaneous emission (ASE), variable stripe length and ASE stability measurements. The results were presented in Figure 3 and supplementary Figure 7.

7. Theoretical analysis

Theoretical analysis that ensures that the experimental values measured are realistic and reasonable
e.g. laser threshold, linewidth, cavity gain-loss, efficiency

☐ Yes
☒ No

- Theoretical work was previously demonstrated in other colloidal nanocrystals.

8. Statistics

Number of devices fabricated and tested

☒ Yes
☐ No

- We characterized 4 different laser devices .

Statistical analysis of the device performance and lifetime (time to failure)

☒ Yes
☐ No

- The ASE signal was stable up to 9.5 hours under continuous pumping above the gain threshold. (Figure 3e).
